# Falls prevention and management for older adults in home care services in Norway: a retrospective patient record review

**DOI:** 10.1007/s41999-025-01224-w

**Published:** 2025-05-04

**Authors:** Rune Solli, Nina Rydland Olsen, Linda Aimée Hartford Kvæl, Kristin Taraldsen, Therese Brovold

**Affiliations:** 1 Faculty of Health Sciences, Department of Rehabilitation Science and Health Technology, OsloMet - Oslo Metropolitan University, Pilestredet 44, 0167 Oslo, Norway; 2 Faculty of Health and Social Sciences, Department of Health and Functioning, Western Norway University of Applied Sciences, Inndalsveien 28, 5063 Bergen, Norway; 3 Norwegian Social Research (NOVA), OsloMet - Oslo Metropolitan University, Oslo, Norway

**Keywords:** Primary care, Older adults, Fall prevention, Fall risk assessment, Retrospective patient record review, Chart review

## Abstract

**Purpose:**

To evaluate Norwegian home care services’ adherence to the World Falls Guidelines 2022 (WFG2022) recommendations on assessment and management of falls among older adults with a history of falling, and to evaluate the degree to which identified fall risk factors were addressed with interventions to prevent falls.

**Methods:**

We conducted a retrospective patient record review of older adults who receive home care services and have a history of falls from four municipal home care service city districts in Oslo, Norway. We collected data from electronic patient records on patient characteristics, the assessed fall risk factors, and implemented interventions to prevent falls. Adherence was analysed as the proportion of patients who received fall prevention care in line with WGF2022 recommendations.

**Results:**

The records of 225 patients were reviewed, of which 157 (70%) patients were between the ages of 70 and 89 years, and 131 (58%) were women. A total of 54 (24%) patients had intermediate fall-risk and 171 (76%) patients had high fall-risk. Of the 54 patients with intermediate fall-risk, 50% received an assessment of balance, gait, or muscle strength, and 22% were subsequently offered exercise. Sixty-one percent of intermediate-risk patients received multifactorial fall risk assessments, and 19% received multifactorial interventions. Exercise was the most used intervention to prevent falls, but it was provided to fewer than half of the patients. Other relevant interventions, such as osteoporosis treatment and medication management, were provided to fewer than 10% of patients.

**Conclusion:**

The results highlight substantial gaps in the comprehensive management of fall prevention where considerably fewer patients received follow-up interventions to prevent falls compared to those who had fall risk factors identified. These results underscore the urgent need for enhanced implementation of fall prevention care within municipal home care services.

**Supplementary Information:**

The online version contains supplementary material available at 10.1007/s41999-025-01224-w.

## Key summary points

**Aim:**

To evaluate Norwegian home care services’ adherence to the World Falls Guidelines 2022 recommendations on assessment and management of falls among older adults with a history of falling, and to evaluate the degree to which identified fall risk factors were addressed with interventions to prevent falls.

**Findings:**

Of the 225 patient records reviewed, 54 older patients had intermediate fall-risk, of which 61% received multifactorial fall risk assessments and 19% received multifactorial interventions. Of the 171 patients with high fall-risk, 70% received multifactorial fall risk assessments, and 40% received multifactorial interventions.

**Message:**

Overall, our results suggest reallocating home care resources from performing multifactorial fall risk assessments on all patients to better addressing identified fall risk factors in high-risk patients.

**Supplementary Information:**

The online version contains supplementary material available at 10.1007/s41999-025-01224-w.

## Introduction

Community-dwelling adults aged 65 years or older with a history of falling have up to three times the risk of falling again [1, 2] and sustaining an injurious fall [3], compared to older adults with no history of falling. Relative to the general population of older adults, recipients of home care have a higher incidence of falls, especially if they have a history of falling [4, 5]. Having experienced a fall in the past year is one of the most important risk factors for future falls [1, 2], which may result in serious consequences including injuries, morbidity, and mortality [6]. Falls also pose a substantial economic burden to society, contributing to up to 1.5% of total health care expenditures [7, 8]. Home care services play a crucial role in fall prevention [9], where healthcare practitioners (HCPs) provide individual and interdisciplinary care to people in their own homes, covering services like home nursing, practical assistance, physiotherapy, occupational therapy, and safety alarms [10]. Successful implementation of effective falls prevention interventions may benefit older recipients of home care by reducing injuries, improving quality of life, and increasing independence [11, 12]. The benefits obtained could reduce the economic burden on society by decreasing healthcare costs associated with fall-related hospitalisations and emergency department visits, reduce the workload on HCPs, and potentially enable more older adults to continue contributing to society [11–13].

The 2022 World guidelines for falls prevention and management for older adults (WFG2022) consolidate earlier research and offer recommendations on fall risk screening, assessment, and effective interventions for fall prevention and management [14]. The WFG2022 categorises fall risk into low, intermediate and high. Older adults with no falls the past year, or with one fall but no gait and balance impairment, are considered at low fall risk and should be offered education about falls prevention and exercise. Older adults with one non-severe fall (no injury requiring medical attention) the past year and impaired gait and balance are considered at intermediate fall risk. For these older adults, it is recommended to offer tailored exercises for balance and muscle strength and advice on fall prevention [14]. Older adults with two or more falls the past year, or with one fall accompanied by injury, frailty, long lie [15], or loss of consciousness, are at high risk of falling. High fall-risk individuals should receive multifactorial fall risk assessments and multifactorial interventions, which must include at least two components, one of which is tailored exercises for balance and muscle strength [14]. The multifactorial interventions should be based on individual risk factors. Older adults presenting with a fall should be asked about the details of the fall, i.e., the frequency of their fall(s), their characteristics, and any consequences. HCPs should assess physical resources (e.g., dependency in activities of daily living), psychological resources (dementia or cognitive impairment), social resources (formal or informal caregivers) and individuals’ perspectives, i.e., goals, beliefs, and priorities, among older adults presenting with a fall [14]. Older adults at high fall-risk should be given the opportunity to co-design the fall prevention plan and should receive a follow-up after 30 to 90 days [14].

Despite the publication of multiple clinical practice guidelines on fall prevention for older adults over the past few decades [14, 16–18], non-adherence remains an issue in primary care [19–22], in emergency departments [23] and in hospitals [24]. For instance, a cross-sectional survey of physiotherapists in Switzerland reported that 62% perform a standardised assessment of fall risk among their older adult patients [25]. Of these, only 14% of physiotherapists working in institutions and 4% of physiotherapists working in private practice carry out systematic fall risk assessment, that is, screen at least 95% of their older adult patients. Although offering exercise to all older adults is recommended [14], earlier patient record reviews conducted in primary care showed that exercise was not prescribed to all older adults, but to 71% [21], 85% [22], and 89% [20], respectively. Additionally, it is recommended that all patients at high fall risk who use fall-risk-increasing drugs should receive a medication intervention [14, 16–18]. Patient record reviews have shown that the percentage of patients who received such an intervention were much lower, for example 21% [20], 22% [22] and 49% [19], respectively. Additionally, a study from primary care found that only 11% of older patients were proactively asked about falls in the previous year by their GP [19]. Ongoing efforts are focused on implementing recommendations for fall prevention in Norwegian municipal home care [26–30]. To our knowledge, no studies have evaluated current fall prevention and management practices against the WFG2022 recommendations among older adults with a history of falling. Therefore, the aim of this study was to evaluate Norwegian home care services’ adherence to the WFG2022 recommendations on assessment and management of falls among older adults with a history of falling, and to evaluate the degree to which identified fall risk factors were addressed with interventions to prevent falls.

## Methods

### Design

We conducted a retrospective, descriptive patient record review of electronically prerecorded patient data [31]. We followed previously published guidance on the conducting and reporting of retrospective record reviews for implementation research [31–33]. The study is part of the research project FALLPREVENT – Implementation of evidence-based fall prevention programs in health care services in Norway [34].

### Context

This study was conducted in the municipal home care services in four city districts of Oslo, Norway. Each of the four participating city districts have more than 20 000 inhabitants [35, 36]. Home care services in Norway consist of nursing, rehabilitation, and practical assistance delivered by multidisciplinary HCPs, as well as the safety alarm service. These services constitute the lowest level of formal care in Norway and are financed mainly by the municipalities [37]. The city districts routinely document health services in Gerica, Oslo municipality’s patient administration system and electronic patient record [38].

In 2021, the city districts introduced a checklist (Online resource 1) based on concurrent evidence from UpToDate [39] to assist HCPs in assessing and managing fall risk in older adults with a fall history. The checklist contained four domains: (i) the individual’s fall risk (e.g., impaired gait or balance, or urinary incontinence), (ii) home risk (e.g., clutter, overfurnishing, or loose carpets in the home), (iii) social network (e.g., participation in activities outside the home), and (iv) assessment of the cause of the patient’s fall. The content of the checklist aligns with the WFG2022 in several areas, including significant overlap in the types of risk factors that should be assessed during a multifactorial fall risk assessment and the types of interventions included in a multifactorial fall prevention intervention [14]. However, the checklist did not include fall risk stratification, as is recommended by the WFG2022 [14]. Instead, it was routine for all city districts to perform multifactorial fall risk assessments and interventions to all older adults who had fallen.

### Criteria for risk stratification and adherence to the WFG2022 recommendations

This section outlines the criteria for fall risk stratification and adherence to the WFG2022 recommendations [14]. Since frailty status was not systematically assessed in the city districts, we substituted frailty status with the activities of daily living (ADL) dependency score, in accordance with recommendations from the Norwegian Directorate of Health [40]. We used a cut-off of three on the Norwegian Individually-based Nursing and Care Statistics (IPLOS) [40] to distinguish between extensive and limited need for assistance with ADL [40, 41]. We assigned intermediate fall-risk to patients with one fall the past year, an ADL dependency score lower than three, and no admission to an institution following the fall. We assigned high fall-risk to patients with two or more falls the past year, with an ADL dependency score of three or higher, or with an admission to an institution following the fall. No patients were classified as low fall risk, as the study focused on older home care recipients with a history of falling [4, 5]. Table 1 lists the WFG2022 recommendations for falls prevention and management for community-dwelling older adults [14] and the criteria used in this study to judge adherence to these recommendations.

**Table 1:** Criteria for adherence to the WFG2022 recommendations

Recommendations	Criteria for adherence: The city districts should have documented …
*Assessments*	
Assess the details of the fall(s), i.e., the frequency, characteristics, and consequences	The frequency (number of falls past year), or characteristics (circumstances, e.g., time, injury, location, cause), or consequences (admissions or visits to institutions following the fall) of the fall, as judged by data collectors
Assess patient’s physical resources after a fall	ADL dependency score
Assess patient’s psychological resources after a fall	An assessment of dementia or cognitive impairment
Assess patient’s social resources after a fall	An assessment of whether the patient had caregivers who could assist
Offer a multifactorial fall risk assessment to patients with high fall risk	An assessment of two or more risk factors for falling listed in the WFG2022 [14], to patients with high fall risk
*Intervention components linked to risk factor assessments*	
Offer physical exercise or physiotherapy if problems with balance, gait, and/or muscle strength, to patients with intermediate fall risk and to patients with high fall risk	(An offer of) physical exercise or of a referral to a physiotherapist, after assessment of balance, gait, or muscle strength
Offer an intervention for footwear and/or feet to high-risk patients with footwear and/or feet issues	(An offer of) an intervention for feet and/or footwear if issues with feet and/or footwear was documented
Offer vision and/or hearing intervention to high-risk patients with impaired vision and/or hearing	(An offer of) an intervention for vision and/or hearing if loss of vision and/or hearing was documented
Offer osteoporosis treatment to patients with osteoporosis and high fall risk	(An offer of) osteoporosis treatment if fall-related fractures during the past 10 years was documented
Offer a medication intervention to patients with polypharmacy and high fall risk	(An offer of) a medication intervention if polypharmacy (use of ≥ 4 prescription medications) was documented
Offer a nutritional intervention to patients with a poor nutritional status and high fall risk	(An offer of) a nutritional intervention if an assessment of unintended weight loss was documented
Offer an environmental intervention to patients with environmental risks and high fall risk	(An offer of) an environmental intervention if an assessment of risk factors in the patient’s home (listed in Online resource 1) was documented
*Multifactorial interventions*	
Offer multifactorial fall prevention interventions to patients with high fall risk	(An offer of) an intervention consisting of two or more intervention components listed in the WFG2022 [14], one of which is physical exercise or a physiotherapist referral, to patients with high fall risk

### Eligibility criteria, study sample and data collection

Eligible patients were adults aged > 65 years with a registered fall who lived in their own home, and who received home care services and/or had a safety alarm. From August to October 2022, 374 older patients in the four city districts were registered with at least one fall. Due to budgetary constraints, we collected data for the first 225 patients who had fallen during the period. The sample was selected from the city districts’ laboratory records and from monthly fall reports, where the city districts routinely documented who and how many older home care recipients had fallen.

Between January and May 2023, the patient records were reviewed by one HCP from each city district, three physiotherapists and one nurse, with knowledge about the city district’s routines for documentation. The steps we took for a thorough and consistent data collection process across city districts are outlined in Table S1 (Online resource 2), adapted from Prusaczyk et al. [33].

We developed a data collection tool (Online resource 3). The tool was piloted on the patient records of five patients, and minor adaptations were made to the tool. The following data related to patient characteristics were collected: age; sex; living situation; the number of falls the past year; minutes of allocated care time per week for home nursing day- and night time; received occupational therapy, physiotherapy and/or practical assistance; allocated care time per week for practical assistance (minutes), admission or visit to an institution following the fall and dependency in ADL [40]. Furthermore, it was registered whether details of the fall had been assessed, including patients’ physical resources, psychological resources, social resources, and whether each risk factor had been assessed and if an intervention had been offered. The extracted data were uploaded to Services for Sensitive Data (TSD) [42], a platform for researchers to securely collect, store and analyse sensitive research data.

### Data analysis

We tabulated patients’ characteristics as the number of observations and percentages for categorical variables and medians and minimum and maximum values for continuous variables, across fall risk groups. Adherence was analysed and reported as the number and proportion of patients who received fall prevention care in accordance with each recommendation (Table 1). All analyses were conducted using Stata, version 18.0, StataCorp, College Station, Texas [43].

### Ethical considerations

The Regional Committees for Medical Research Ethics – South East Norway granted an exemption from the duty of confidentiality to access and analyse relevant data to achieve this study’s objectives (ref. no. 520104). Therefore, informed consent was not sought. This study was registered with the Norwegian agency for shared services in education and research (Sikt) (ref. no. 552554). A data protection impact assessment (DPIA) was conducted in collaboration with Sikt and Oslo Metropolitan University`s (OsloMet’s) data protection officer to safeguard the privacy of the patients. We conducted a risk assessment to maintain the confidentiality, integrity, and availability of data from the patients. Next, we signed a project cooperation agreement as well as a data transfer agreement (DTA) with each city district, and data were stored and analysed securely in TSD [42]. The collected data were de-identified using a key code list and securely stored at OsloMet. Patients were informed about the project and were given the choice to withdraw at any time without consequence.

## Results

### Patient characteristics

The patient records of 225 older adults were included in this study. Of these, 157 (70%) patients were between the ages of 70–89, and 131 (58%) were women. A total of 54 (24%) patients were stratified to intermediate fall-risk, and 171 (76%) to high fall-risk. Patients’ characteristics are shown in Table 2.

**Table 2:** Patient characteristics

Variable	Missing *n* (%)	All patients *n* = 225	Intermediate fall risk, *n* = 54	High fall risk, *n* = 171
*n* (%)				
Age, years	3 (1)			
65–69		10 (5)	0 (0)	10 (6)
70–79		65 (29)	16 (30)	49 (29)
80–89		92 (41)	23 (43)	69 (40)
90–99		53 (24)	14 (26)	39 (23)
≥ 100		2 (1)	0 (0)	2 (1)
Women		131 (58)	39 (72)	92 (54)
Living situation (living alone)		182 (81)	42 (78)	140 (82 )
Care services		224 (99)	54 (100)	170 (99)
Home nursing (daytime, evenings, weekends)		182 (81)	38 (70)	144 (84)
Home nursing (nighttime)		28 (12)	4 (7)	24 (14)
Practical assistance		139 (62)	29 (54)	110 (64)
Safety alarm		109 (48)	27 (50)	82 (48)
Transport service cards		113 (50)	26 (48)	87 (51)
Occupational therapy		59 (26)	12 (22)	47 (27)
Physiotherapy		34 (15)	7 (13)	27 (16)
*Median (min–max)*				
ADL score*	6 (3)	2.4 (1.5–4.5)	2.1 (1.5–3)	2.7 (1.5–4.5)
Allocated care time per week, minutes^†^		259 (5–2 486)	150 (14–1 680)	306 (5–2 486)
Allocated time with practical assistance per week, minutes		30 (1–195)	37 (20–105)	30 (1–195)
Number of diagnoses	43 (19)	3 (0–18)	2 (0–14)	4 (0–18)
Number of medicines	37 (16)	8 (0–34)	7 (0–17)	9 (0–34)

*IPLOS functioning variables from the Norwegian Individually-based Nursing and Care Statistics registry (score from one to five, higher score indicating lower functioning) [40]

^†^Includes home nursing daytime, home nursing nighttime, physiotherapy, occupational therapy

### Assessment of risk factors and follow-up with individualised interventions

**Fig. 1:**
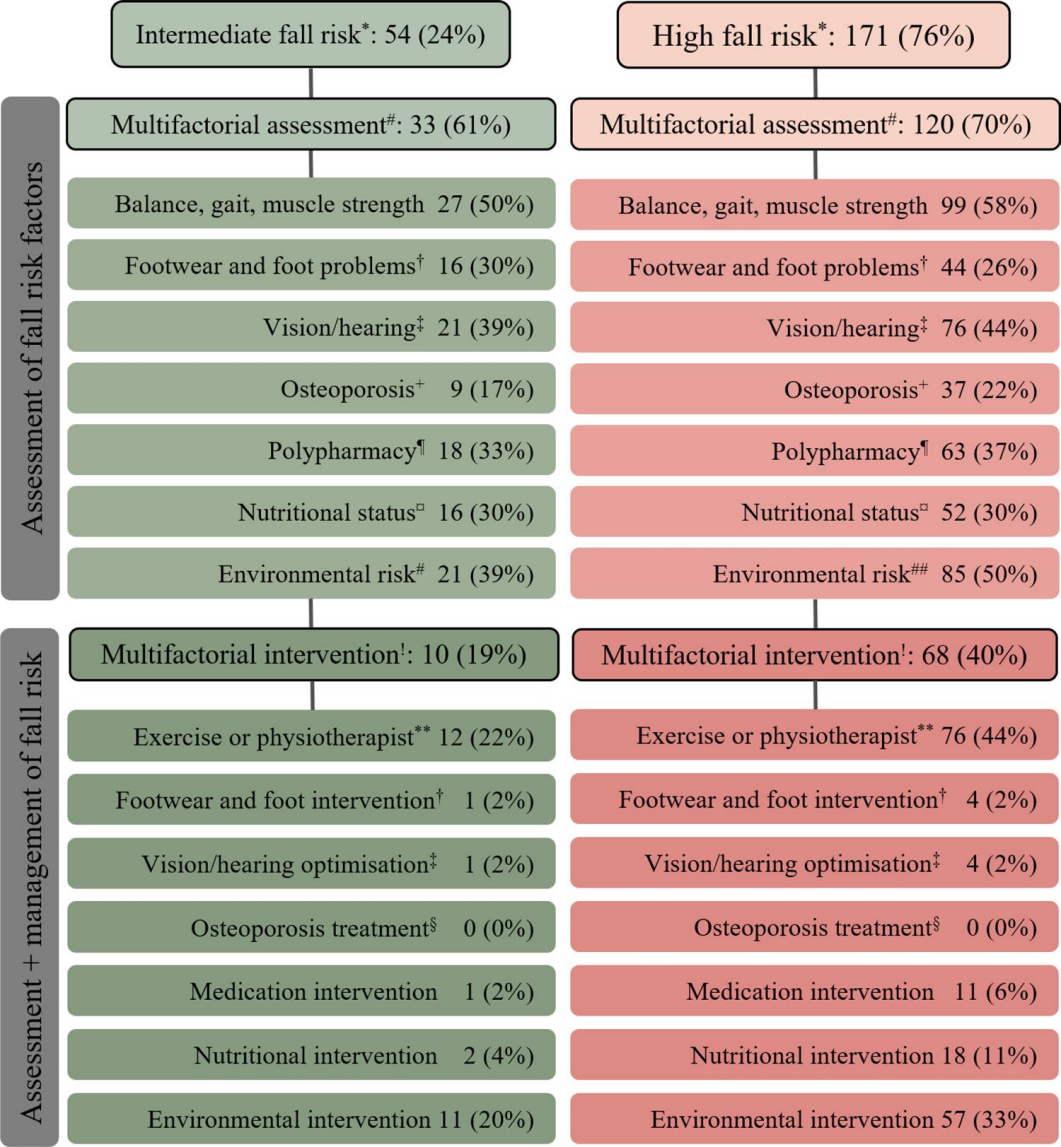
Fall risk stratification and assessment and management of fall risk factors. The figure shows the number and proportion of patients who were stratified into intermediate or high fall risk, and of those, how many received fall risk assessments, or both fall risk assessments and the corresponding interventions. *Intermediate fall risk: 1 fall past 12 months and ADL score < 3 and no admission to an institution following the fall. High fall risk: ≥ 2 falls past 12 months or ADL score ≥ 3 or an admission to an institution following the fall. ^#^An assessment of ≥ 2 modifiable fall risk factors. ^!^An intervention consisting of ≥ 2 components, one of which was exercise or referral to a physiotherapist. **An intervention including physical exercise or referral to physiotherapist. ^†^Assessment of issues with feet or footwear. Management included advice about footwear and ordering new shoes, or a referral to a pedicurist. ^‡^May include advice about glasses, or referral to optician or ophthalmologist. ^+^Assessment of fall-related fractures during the past 10 years. ^§^No information about osteoporosis assessment or treatment available in the patient records. ^¶^Use of four or more prescription medications. ^¤^Assessment of unintended weight loss. ^##^Assessment of risk factors in the patient’s home

Among the 54 patients with intermediate fall-risk, 27 (50%) received an assessment of balance, gait, or muscle strength, and 12 (22%) of these were offered exercise or physiotherapy. Thirty-three (61%) intermediate fall-risk patients received multifactorial fall risk assessments, and 10 (19%) subsequently received multifactorial interventions. Figure 1 shows the number and proportion of patients who received fall risk assessments or both assessments and interventions. For patients with intermediate fall-risk, a median of three fall risk factors were assessed per patient, and a median of one fall risk factor was followed up with an intervention (Table S2, Online resource 4).

Among the 171 patients with high fall-risk, 120 (70%) received multifactorial fall risk assessments, and 68 (40%) also received multifactorial interventions. The most frequently initiated intervention was exercise or a physiotherapist referral. A total of 99 (58%) high fall-risk patients received an assessment of balance, gait, or muscle strength, and 76 (44%) of these were offered exercise or physiotherapy (Fig. 1). Environmental risk was documented for 85 (50%) high fall-risk patients, and 57 (33%) of these patients received an environmental intervention. Polypharmacy was documented for 37 (22%) high fall-risk patients, and 11 (6%) of these patients subsequently received a medication intervention. A median of seven risk factors were assessed for each high fall-risk patient, and a median of two risk factors were followed up with an intervention (Table S2, Online resource 4). Adherence to the WFG2022 recommendations is shown in detail in Online resource 4.

### Assessment of the cause, characteristics, and consequences of the falls

HCPs documented the cause of the fall for 35 (65%) and 126 (74%) intermediate and high fall-risk patients, respectively. A description of the characteristics of the fall was documented in the records for 35 (65%) and 135 (79%) intermediate- and high-risk patients, respectively. Data on admission or visit to an institution, e.g., a hospital or an emergency department, following the fall was available for 201 (89%) patients: of these, there were no admission after 159 (79%) falls, admission to hospital after 21 (11%) falls, admission to the emergency department after 17 (7%) falls, admission to a 24-h municipal acute unit after 3 (1%) falls, and admission to intermediate care after 1 (1%) fall.

### Assessment of the older adults’ resources

ADL dependency scores (physical resources) were assessed for all but six patients (Table 2). Assessment of dementia or cognitive impairment (psychological resources) was documented in 13 (24%) and 62 (36%) intermediate and high fall risk patients, respectively. Assessment of whether the patient had caregivers who could assist (social resources) was documented in 27 (50%) of intermediate-risk patients and 86 (50%) of high-risk patients.

## Discussion

In this study, we retrospectively reviewed patient records of 225 older adults to evaluate the extent to which they were offered the recommended fall prevention assessments and interventions after a fall. Our patients were identified with intermediate (24%) or high fall-risk (76%). Half of the intermediate-risk patients received an assessment of balance, gait, and muscle strength, and 22% received exercise or physiotherapy. Two thirds of the high-risk patients received a multifactorial fall risk assessment, however only 40% received multifactorial interventions based on the identified risk factors.

In this patient record review, 70% of high-risk patients received multifactorial fall risk assessments. Previous record reviews found even lower rates of multifactorial assessment of risk factors for falls in primary care settings [19–22]. For instance, Eckstrom et al. [22] reported that 64% of the high-risk patients received multifactorial fall risk assessments. Phelan et al. [20] found that, during the period of 2010 to 2012, 54% of high-risk patients received at least half of the recommended fall risk factor assessments. However, even earlier chart review studies found that the performance of most fall risk assessments was 50% or lower [19, 21]. A potential explanation for this apparent improvement in assessment rates over time include the publication of systematic reviews and updated guidelines on fall prevention, and an increased focus on the implementation of recommendations for fall prevention in recent years [12, 16]. Thus, both this study and earlier studies suggest that a substantial proportion of older adults with high fall-risk do not receive the recommended assessments or interventions, potentially leading to more falls and injuries [12, 14].

In this study, 40% of high-risk patients received multifactorial interventions to prevent falls. Previous patient record reviews reported a higher proportion of patients receiving multifactorial interventions than in this study, ranging between 73 and 64% [20, 22]. A possible explanation for the more frequent prescribing of multifactorial fall prevention interventions in previous patient record reviews is the types of fall prevention interventions evaluated. For instance, previous studies covered interventions for assistive devices [20] and for low vitamin D and orthostatic hypotension [20, 22], in addition to other risk factors such as impaired gait, vision problems, and issues with feet or footwear. In comparison, this study did not cover interventions specifically for assistive devices, low vitamin D, or orthostatic hypotension.

We found that 19% of the intermediate-risk patients received multifactorial fall risk assessments and interventions, although it is not recommended that this group of patients receive this type of assessment [14]. This finding is not surprising, considering that the city districts’ procedures and the checklist they used indicated that all older adults with a fall should receive multifactorial fall risk assessments and interventions. However, for the future, the time and resources spent providing multifactorial interventions to intermediate-risk patients can be directed towards high-risk patients in line with the WGF2022 recommendations [14].

A combination of balance and muscle strength exercise has been shown to be the single most effective fall prevention intervention for community-dwelling older adults [12] and is recommended to be offered to all older adults [14]. In our study, exercise or a physiotherapist referral was the most frequently prescribed intervention component in both risk-groups, however, surprisingly only 22% of intermediate-risk patients and 44% of high-risk patients received this intervention. In comparison, results from earlier patient record reviews in primary care showed that exercise was one of the most frequently prescribed interventions to prevent falls, prescribed to 71% [21], 85% [22], and 89% [20] of patients, respectively.

Medication review is a recommended component of multifactorial fall prevention interventions that have been shown to reduce the rate of falls [14, 44]. Only 6% of the high fall-risk patients in our study received a medication intervention after documentation of polypharmacy. Earlier patient record reviews reported that the number of patients receiving a medication intervention for their use of fall-risk-increasing drugs were 21% [20], 22% [22] and 49% [19]. Polypharmacy is common in older adults [45] and is important to address given its association with increased risk of falls [2].

The complex nature of fall prevention, with a multitude of risk factors and the need for multidisciplinary management [14], suggest that patients would benefit from improved collaboration between HCPs in primary care [14, 46, 47]. One possible explanation for the low adherence to medication interventions in our study could be insufficient collaboration between home care services and GPs, as reported in a meta-ethnography of Norwegian studies [48]. One solution could be to implement routine fall prevention activities as a collaborative effort, such as in a recent randomised trial from Australia where the GP or practice nurse reviewed fall risk checklists completed by older adults, conducted fall risk assessments, and offered tailored interventions, including referrals to allied health professionals where appropriate [46]. Compared to control group GPs, GPs who applied this intervention were more likely to engage in fall prevention activities [46]. Similarly, the American Geriatrics Society recommends a team-based approach to fall prevention in the community, where HCPs from various disciplines are identified and involved, to increase patient adherence and improve outcomes [49].

Our study highlights that assessing and identifying fall risk does not guarantee that patients are offered interventions to prevent falls. For high-risk patients, the disparity between the high number receiving multifactorial fall risk assessments (70%) and the low number receiving interventions (40%) imply that there is still a need to improve adherence to recommendations for community-dwelling older adult recipients of home care services. As the city districts currently do not use risk stratification, this should be amended in line with the recommendations [14]. Thus, HCPs should incorporate routines tailored to the patient’s level of fall risk. Incorporating routine fall risk stratification, assessments, and individualised management and interventions based on identified fall risk factors may help HCPs proactively address fall risk before serious fall injuries occur, potentially reducing hospitalisation rates [12, 14]. Furthermore, a future implementation study in this context should target the link between assessments and interventions, that is, how best to initiate interventions based on identified fall risk factors. Knowledge on the relationship between assessments and interventions may enhance the uptake of the evidence on fall prevention in the city districts [14, 44]. Such a study should include outcomes such as fall injuries, hospitalisations or all-cause mortality, as well as the delivery of tailored interventions based on identified fall risk factors, to demonstrate the impact of the intervention.

The municipalities should consider incorporating routine frailty assessments [14]. Being frail increases an older adults’ risk of falling [50], and frailty assessment is a recommended component of fall risk stratification [14]. Either previously identified frailty or a positive result on a validated instrument, such as the Clinical Frailty Scale [51], may be used. For intermediate-risk patients who received more detailed fall prevention care than what was recommended, the routines should be amended, as the WFG2022 do not recommend multifactorial assessments and interventions for intermediate-risk patients [14]. For low-risk patients, the home care services lack a method for identifying this group of older adults, as they do not stratify fall risk. A future study should explore methods for identifying older adults at low fall risk, such as automatically detecting those with no registered fall in the past 12 months in their patient records. Future research should also explore ways of offering minimal yet effective interventions to low-risk patients in this context, such as educational programs and materials reinforcing health promotion and the prevention of falls and fractures, exercise and avoidance of being sedentary for general health and fall prevention, and annual reassessment [14].

A strength of this study was the thorough data collection conducted by HCPs with years of experience in both fall prevention practice and in use of the municipality’s electronic patient record system, using a piloted data collection tool. Still, we cannot exclude the possibility that some data may be lacking, either because it was overlooked in the patient records or because it was not documented in the patient records to begin with.

A limitation of our study is that we used convenience sampling for the recruitment of city districts. Consequently, neither city districts nor patients were selected at random. However, we included the first 225 registered fallers during the study period, suggesting that no fallers were excluded based on the documentation of the fall prevention activities documented in their patient records. This suggests that the sample of electronic patient records was representative of the population in similarly sized city districts and municipalities. The four city districts that participated in this study are large with more than 20 000 inhabitants [35, 36]. Large municipalities have higher degrees of specialisation in their care services and higher formal competencies in their workforce than smaller municipalities [37]. Hence, small- to medium-sized municipalities may lack the level of specialisation and formal competency to that of the participating city districts, and as a result, their adherence to fall prevention recommendations may differ. This may limit the generalisability of our results to smaller municipalities. Another limitation is that we did not have access to GPs’ patient records, which may have contained information about fall preventive care that was not reflected in the electronic patient records of the city districts. For instance, osteoporosis treatment and medication interventions may have been provided to patients by their GP without being documented in the city districts’ electronic patient records.

## Conclusions

In this study, we conducted a patient record review of 225 older adults from the municipal home care services, all classified as either intermediate- or high-risk for falls. The number of patients who received interventions to prevent falls was considerably lower than those identified with fall risk factors. Exercise was the most used intervention but was offered to fewer than half of the patients. Multiple patients with intermediate fall-risk were provided with multifactorial fall risk assessments and interventions, which is not recommended and may be unnecessarily complicated. Our results indicate a need for better prescribing of fall prevention interventions across all risk factors in the high-risk group, especially for osteoporosis, medications, vision, hearing, feet, and nutrition. Overall, our results suggest that the home care services should redirect resources from conducting multifactorial fall risk assessments for all patients to better intervening on identified fall risk factors in high-risk patients.

## Supplementary Information

Below is the link to the electronic supplementary material.

Supplementary file1 (PDF 214 KB) Checklist for fall prevention home visits

Supplementary file2 (PDF 160 KB) Steps to promote thorough and consistent data collection

Supplementary file3 (PDF 428 KB) Data collection tool

Supplementary file4 (PDF 158 KB) Detailed results

## Data Availability

The data presented in this study are not readily available because of privacy restrictions.

## References

[1] Li Y, Hou L, Zhao H, Xie R, Yi Y, Ding X (2023) Risk factors for falls among community-dwelling older adults: a systematic review and meta-analysis. Front Med (Lausanne). 10.3389/fmed.2022.1019094 36687461 10.3389/fmed.2022.1019094 PMC9853191

[2] Shao L, Shi Y, Xie X-Y, Wang Z, Wang Z-A, Zhang J-E (2023) Incidence and Risk factors of falls among older people in nursing homes: systematic review and meta-analysis. J Am Med Dir Assoc. 10.1016/j.jamda.2023.06.002 37433427 10.1016/j.jamda.2023.06.002

[3] Poss JW, Hirdes JP (2016) Very frequent fallers and future fall injury: continuous risk among community-dwelling home care recipients. J Aging Health 28:587–599. 10.1177/0898264315599941 26270720 10.1177/0898264315599941

[4] Fletcher PC (2004) Restriction in activity associated with fear of falling among community-based seniors using home care services. Age Ageing 33:273–279. 10.1093/ageing/afh077 15082433 10.1093/ageing/afh077

[5] Manis DR, McArthur C, Costa AP (2020) Associations with rates of falls among home care clients in Ontario, Canada: a population-based, cross-sectional study. BMC Geriatr 20:80. 10.1186/s12877-020-1483-6 32106824 10.1186/s12877-020-1483-6 PMC7047389

[6] James SL, Lucchesi LR, Bisignano C, Castle CD, Dingels ZV, Fox JT et al (2020) The global burden of falls: global, regional and national estimates of morbidity and mortality from the Global Burden of Disease Study 2017. Inj Prev 26:i3-11. 10.1136/injuryprev-2019-043286 31941758 10.1136/injuryprev-2019-043286 PMC7571347

[7] Kinge JM, Dieleman JL, Karlstad Ø, Knudsen AK, Klitkou ST, Hay SI et al (2023) Disease-specific health spending by age, sex, and type of care in Norway: a national health registry study. BMC Med 21:201. 10.1186/s12916-023-02896-6 37277874 10.1186/s12916-023-02896-6 PMC10243068

[8] Heinrich S, Rapp K, Rissmann U, Becker C, König H-H (2010) Cost of falls in old age: a systematic review. Osteoporos Int 21:891–902. 10.1007/s00198-009-1100-1 19924496 10.1007/s00198-009-1100-1

[9] Vieira ER, Palmer RC, Chaves PHM (2016) Prevention of falls in older people living in the community. BMJ. 10.1136/bmj.i1419 27125497 10.1136/bmj.i1419

[10] Thomé B, Dykes A, Hallberg IR (2003) Home care with regard to definition, care recipients, content and outcome: systematic literature review. J Clin Nurs 12:860–872. 10.1046/j.1365-2702.2003.00803.x 14632979 10.1046/j.1365-2702.2003.00803.x

[11] Olij BF, Ophuis RH, Polinder S, van Beeck EF, Burdorf A, Panneman MJM et al (2018) Economic evaluations of falls prevention programs for older adults: a systematic review. J Am Geriatr Soc 66:2197–2204. 10.1111/jgs.15578 30325013 10.1111/jgs.15578

[12] Gillespie LD, Robertson MC, Gillespie WJ, Sherrington C, Gates S, Clemson LM et al (2012) Interventions for preventing falls in older people living in the community. Cochrane Database Syst Rev. 10.1002/14651858.CD007146.pub3 22972103 10.1002/14651858.CD007146.pub3 PMC8095069

[13] Hartholt KA, van Beeck EF, Polinder S, van der Velde N, van Lieshout EMM, Panneman MJM et al (2011) Societal consequences of falls in the older population: injuries, healthcare costs, and long-term reduced quality of life. J Trauma Injury Infect Crit Care 71:748–753. 10.1097/TA.0b013e3181f6f5e5 10.1097/TA.0b013e3181f6f5e5 21045738

[14] Montero-Odasso M, van der Velde N, Martin FC, Petrovic M, Tan MP, Ryg J et al (2022) World guidelines for falls prevention and management for older adults: a global initiative. Age Ageing 51:1–36. 10.1093/ageing/afac205 10.1093/ageing/afac205 PMC9523684 36178003

[15] Kubitza J, Schneider IT, Reuschenbach B (2023) Concept of the term long lie: a scoping review. Eur Rev Aging Phys Activ 20:16. 10.1186/s11556-023-00326-3 10.1186/s11556-023-00326-3 PMC10463813 37644386

[16] Updated American Geriatrics Society/British Geriatrics Society Clinical Practice Guideline for prevention of falls in older persons and recommendations. American Geriatrics Society; British Geriatrics Society, 2010. 10.1111/j.1532-5415.2010.03234.x 21226685

[17] NICE (2013) Falls: Assessment and prevention of falls in older people (NICE clinical guideline CG161). United Kingdom.

[18] Sundhedsstyrelsen (2018) Nationale kliniske retningslinjer for forebyggelse af fald. 1st ed. København: Sundhedsstyrelsen

[19] Askari M, Eslami S, van Rijn M, Medlock S, Moll van Charante EP, van der Velde N et al (2016) Assessment of the quality of fall detection and management in primary care in the Netherlands based on the ACOVE quality indicators. Osteopor Int 27:569–76. 10.1007/s00198-015-3235-6 10.1007/s00198-015-3235-6 PMC4740558 26194490

[20] Phelan EA, Aerts S, Dowler D, Eckstrom E, Casey CM (2016) Adoption of evidence-based fall prevention practices in primary care for older adults with a history of falls. Front Public Health. 10.3389/fpubh.2016.00190 27660753 10.3389/fpubh.2016.00190 PMC5014854

[21] Rubenstein LZ, Solomon DH, Roth CP, Young RT, Shekelle PG, Chang JT et al (2004) Detection and management of falls and instability in vulnerable elders by community physicians. J Am Geriatr Soc 52:1527–1531. 10.1111/j.1532-5415.2004.52417.x 15341556 10.1111/j.1532-5415.2004.52417.x

[22] Eckstrom E, Parker EM, Lambert GH, Winkler G, Dowler D, Casey CM (2017) Implementing STEADI in academic primary care to address older adult fall risk. Innov Aging. 10.1093/geroni/igx028 29955671 10.1093/geroni/igx028 PMC6016394

[23] Lillevang-Johannsen M, Grand J, Lembeck M, Giger A-K, Drozdowska J, Zajworoniuk-Wlodarczyk J et al (2017) Falls in elderly patients are not treated according to national recommendations. Dan Med J 64:5A5418 29115205

[24] McEwan H, Baker R, Armstrong N, Banerjee J (2018) A qualitative study of the determinants of adherence to NICE falls guideline in managing older fallers attending an emergency department. Int J Emerg Med 11:33. 10.1186/s12245-018-0192-9 30022394 10.1186/s12245-018-0192-9 PMC6051952

[25] Duc M, Mittaz Hager A-G, Zemp D, Roulet G, Bridel A, Hilfiker R (2023) Current practices of physiotherapists in Switzerland regarding fall risk-assessment for community-dwelling older adults: a national cross-sectional survey. F1000Res 11:1–41. 10.12688/f1000research.73636.2 10.12688/f1000research.73636.2 PMC10733665 38131051

[26] Linnerud S, Kvæl LAH, Bjerk M, Taraldsen K, Skelton DA, Brovold T (2024) Feasibility of an implementation strategy for preventing falls in homecare services. Implement Sci Commun 5:1–14. 10.1186/s43058-024-00615-7 39030646 10.1186/s43058-024-00615-7 PMC11264773

[27] Solli R, Kvæl LAH, Olsen NR, Brovold T (2025) Evaluation of content validity and feasibility of the World Falls Guidelines’ three key questions to identify falls among older adult users of home care services in Norway. BMC Health Serv Res 25:1–13. 10.1186/s12913-025-12606-y 40148859 10.1186/s12913-025-12606-y PMC11948927

[28] Bjerk M, Flottorp SA, Pripp AH, Øien H, Hansen TM, Foy R et al (2024) Tailored implementation of national recommendations on fall prevention among older adults in municipalities in Norway (FALLPREVENT trial): a study protocol for a cluster-randomised trial. Implement Sci 19:5. 10.1186/s13012-024-01334-2 38273325 10.1186/s13012-024-01334-2 PMC10811923

[29] Linnerud S, Bjerk M, Olsen NR, Taraldsen K, Brovold T, Kvæl LAH (2024) Managers’ perspectives on their role in implementing fall prevention interventions: a qualitative interview study in Norwegian homecare services. Front Health Serv 4:1–11. 10.3389/frhs.2024.1456028 10.3389/frhs.2024.1456028 PMC11467783 39399444

[30] Schelbred A-B, Smedshaug G, Haugen IK, Nordvik JE, Belander O, Melby AKI (2024) Fallforebygging hos eldre - Nasjonale faglige råd. Oslo

[31] Vassar M, Matthew H (2013) The retrospective chart review: important methodological considerations. J Educ Eval Health Prof 10:12. 10.3352/jeehp.2013.10.12 24324853 10.3352/jeehp.2013.10.12 PMC3853868

[32] Gregory BH, Van Horn C, Kaprielian VS (2008) Eight steps to a chart audit for quality. Fam Pract Manag 15(7):A3–A8 18763679

[33] Prusaczyk B, Fabbre V, Carpenter CR, Proctor E (2018) Measuring the delivery of complex interventions through electronic medical records: challenges and lessons learned. EGEMs Gener Evid Methods Improve Patient Outcomes 6:10. 10.5334/egems.230 10.5334/egems.230 PMC6078114 30094282

[34] FallPrevent: Implementation of evidence-based fall prevention interventions in the health care services: quality, competency, and effectiveness. OsloMet 2021. https://www.oslomet.no/forskning/forskningsprosjekter/fallprevent.

[35] Bydelsfakta - Statistikk om befolkning, levekår og boforhold. Bydelsfakta 2024. https://bydelsfakta.oslo.kommune.no/ (Accessed June 12, 2024).

[36] Facts about the population - how many lives in Norway? Statistics Norway 2023. https://www.ssb.no/befolkning/faktaside/befolkningen (Accessed January 16, 2024).

[37] Sogstad M, Hellesø R, Skinner MS (2020) The development of a new care service landscape in Norway. Health Serv Insights 13:117863292092222. 10.1177/1178632920922221 10.1177/1178632920922221 PMC7285946 32565676

[38] Gerica electronic health record and patient administration system. Documaster 2024. https://www.documaster.com/integrasjon-gerica (Accessed May 3, 2024).

[39] Kiel DP. Falls: Prevention in community-dwelling older persons. UpToDate 2021. https://www.uptodate.com/contents/falls-prevention-in-community-dwelling-older-persons#subscribeMessage (Accessed August 31, 2024).

[40] Registrering av helse- og omsorgsdata i kommunen: Funksjonsvariablene. Helsedirektoratet 2018. https://www.helsedirektoratet.no/veiledere/registrering-av-iplos-data-i-kommunen/funksjonsvariablene (Accessed March 4, 2024).

[41] Anbefaling fra arbeidsgruppe revidering av IPLOS samlemål: Rapport fra Arbeidsgruppe nedsatt av Helsedirektoratet. Rapport IS-1831. Oslo, Norway: 2010

[42] UiO. Tjenester for Sensitive Data (TSD). Universitetet i Oslo 2022. https://www.uio.no/tjenester/it/forskning/sensitiv/.

[43] StataCorp. Stata Statistical Software: Release 18. College Station, TX 2023

[44] Hopewell S, Adedire O, Copsey BJ, Boniface GJ, Sherrington C, Clemson L et al (2018) What works better for community-dwelling older people at risk to fall?: A meta-analysis of multifactorial versus physical exercise-alone interventions. Cochrane Database Syst Rev. 10.1002/14651858.CD012221.pub2 30035305 10.1002/14651858.CD012221.pub2 PMC6513234

[45] Delara M, Murray L, Jafari B, Bahji A, Goodarzi Z, Kirkham J et al (2022) Prevalence and factors associated with polypharmacy: a systematic review and meta-analysis. BMC Geriatr 22:601. 10.1186/s12877-022-03279-x 35854209 10.1186/s12877-022-03279-x PMC9297624

[46] Clemson L, Mackenzie L, Lovarini M, Roberts C, Poulos R, Sherrington C et al (2024) Integrated solutions for sustainable fall prevention in primary care: a pragmatic hybrid-type 2 mixed methods implementation and effectiveness study. Front Public Health. 10.3389/fpubh.2024.1446525 39703488 10.3389/fpubh.2024.1446525 PMC11656318

[47] Gillespie LD, Robertson MC, Gillespie WJ, Sherrington C, Gates S, Clemson LM et al (2012) Interventions for preventing falls in older people living in the community. Cochrane Database Syst Rev 2012:1–366. 10.1002/14651858.CD007146.pub3 10.1002/14651858.CD007146.pub3 PMC8095069 22972103

[48] Steihaug S, Johannessen A-K, Ådnanes M, Paulsen B, Mannion R (2016) Challenges in achieving collaboration in clinical practice: the case of norwegian health care. Int J Integr Care. 10.5334/ijic.2217 28435416 10.5334/ijic.2217 PMC5351059

[49] Johnson TM, Vincenzo JL, De Lima B, Casey CM, Gray S, McMahon SK et al (2025) Updating STEADI for primary care: recommendations from the American Geriatrics Society Workgroup. J Am Geriatr Soc. 10.1111/jgs.19378 39887356 10.1111/jgs.19378 PMC12303747

[50] Yang Z-C, Lin H, Jiang G-H, Chu Y-H, Gao J-H, Tong Z-J et al (2023) Frailty is a risk factor for falls in the older adults: a systematic review and meta-analysis. J Nutr Health Aging 27:487–495. 10.1007/s12603-023-1935-837357334 10.1007/s12603-023-1935-8

[51] Rockwood K (2005) A global clinical measure of fitness and frailty in elderly people. Can Med Assoc J 173:489–495. 10.1503/cmaj.050051 16129869 10.1503/cmaj.050051 PMC1188185

