# Supplementary material for: Falls prevention and management for older adults in home care services in Norway: a retrospective patient record review
Source: Eur Geriatr Med. 2025 May 4;16(3):1013–23. doi: 10.1007/s41999-025-01224-w (PMC12174202; doi:10.1007/s41999-025-01224-w)
Supplement: Supplementary file 1 — Supplementary file1 (PDF 214 KB) Checklist for fall prevention home visits [file 41999_2025_1224_MOESM1_ESM.pdf]

Online resource 1. Checklist for fall prevention home visits.

Falls prevention and management for older adults in home care services in Norway: A retrospective patient record review

European Geriatric Medicine

- Rune Solli, Department of Rehabilitation Science and Health Technology, Faculty of Health Sciences, OsloMet - Oslo Metropolitan University, Oslo, Norway. E-mail: RuneSoll@OsloMet.no.
- Nina Rydland Olsen.
- Linda Aimée Hartford Kvæl.
- Kristin Taraldsen.
- Therese Brovold.

Sjekkliste[1] fallforebyggende hjemmebesøk

Sjekklisten gjennomført, dato: Gerica id.nr:

1 Individets risiko

**1. 1**	Beskriv fallhendelsen, konsekvenser av fallet?		
		Ja /nei	Hvis ja, forklar
**1.2**	Akutt sykdom eller funksjonssvikt?<br>Ved behov benytt ABCDE/ NEWS/SAFE*		
**1.3**	Svimmelhet/ besvimelser i forbindelse med fall?		
**1.4**	Fall flere ganger siste tre måneder?<br>Brudd i forbindelse med fall siste 10 år?		
**1.5**	Sykdommer som kan øke fallrisiko?		
**1.6**	Demens eller redusert kognitiv funksjon?		
**1.7**	Nedsatt syn og hørsel?		
**1.8**	Ufrivillig vektnedgang siste seks måneder?		
**1.9**	Hyppige toalettbesøk om natta?		
**1.10**	Bruk av 4 eller flere reseptbelagte medikamenter daglig?		
**1.11**	Balanse- og eller gangproblemer?		
**1.12**	Er vedkommende i stand til å reise seg fra en stol? Be personen reise seg fra en kjøkkenstol uten å bruke armene		
**1.13**	Problemer med føtter / sko/ smerter/ hevelse i beina?		
**1.14**	Alkohol-, rusmiddelbruk?		

[1] Sjekkliste oktober 2021, tar utgangspunkt i Fallrisiko hos eldre, sjekkliste - NHI.no, Up to date, erfaringer, og rutiner OK. Utarbeidet i samarbeid mellom representanter fra; OsloMet, Skadelegevakt OUS og Oslo kommune.

**2**	**Risiko i hjemmet**		
**2.1**	Er boligen rotete, overmøblert, løse tepper, ledninger, høye terskler, dårlig belysning?		
**2.2**	Trygt baderom inkl. adkomst?		
**2.3**	Trygt inngangsparti inne og ute? Glatte trapper/ gulv?		
**2.4**	Annet?		
**3**	**Sosialt nettverk**		
**3.1**	Pårørende/ venner som kan bidra?		
**3.2**	Deltakelse i aktiviteter utenfor hjemmet?		
	**Vurdering årsak for hvorfor bruker faller**	**Forslag til tiltak?**	
**1**			
**2**			
**3**			
	Burde pårørende involveres og bruker samtykker?		
	Diskutert tverrfaglig dato:	Fastlege kontaktet?	

*Avhengig av kompetanse, alle skal kunne gjennomføre ABCDE

Sjekklisten benyttes for vurdering av personer som har falt. Kan også benyttes forebyggende for å kartlegge fallrisiko for brukere i tjenestene. Kartleggingen gjennomføres av medarbeidere med helsefaglig utdanning. **Diskuteres i etterkant tverrfaglig** for å komme fram til aktuelle oppfølgingstiltak. Spørsmålet «hva er viktig for deg?» står ikke i sjekklisten, men hva som er viktig for personen vil alltid være avgjørende når tjenester og tiltak iverksettes. Sikre informasjon til fastlege om fall, funn og tiltak. IPLOS ADL skal alltid oppdateres ved endring. Se forøvrig rutine Eqs (lenke kommer) og Gerica manual.

	**Risiko hos bruker**	**Eksempler på tiltak, faglig skjønn må ligge til grunn**
**1.1**	Beskriv fallhendelsen, når, hvor, hvordan samt konsekvenser av fallet?	Viktig for forståelse av hva som har skjedd, alvorlighetsgrad og konsekvenser for personen.
**1.2**	Akutt sykdom eller funksjonssvikt?<br>Ved behov benytt ABCDE/ NEWS/Safe*	Vurder alvorlighetsgrad i samråd med ansvarsvakt. V/ behov kontakt legevakt, fastlege. Hvis alvorlighetsgraden ikke er så stor, diskuter tverrfaglig, obs ortostatisk blodtrykk, hjerterytme, evnt urinprøve.
**1.3**	Svimmelhet/ besvimelser i forbindelse med fall?	
**1.4**	Fall flere ganger siste tre måneder?<br>Brudd i forbindelse med fall siste 10 år?	Ved endring i funksjon, henvis fastlege for utredning av årsak. Brudd: Henvis fastlege for osteoporosebehandling dersom det mangler.
**1.5**	Sykdommer som kan øke fallrisiko?	Vurder endring i funksjon og aktuelle tiltak.
**1.6**	Demens eller redusert kognitiv funksjon?	Er personen tilstrekkelig utredet, tiltak iverksatt, hjemmet tilrettelagt, ernæring/væske tilstrekkelig?
**1.7**	Nedsatt syn og hørsel?	Vurder optiker eller henvisning fra fastlege til øyelege. Vurder at tiltak ved nedsatt hørsel er tilstrekkelige. Kontakt ergoterapeut for tilrettelegging.
**1.8**	Ufrivillig vekttap siste 6 måneder?	Kartlegg årsaker til vekttap. Følg Ernæringstrappen for valg av relevante tiltak. Henvis ev fastlege/kef
**1.9**	Hyppige toalettbesøk om natta?	Diskuter årsak og ved behov iverksett tiltak (henvis fastlege /ergoterapeut)

**1.10**	Bruk av 4 eller flere reseptbelagte medikamenter daglig? (obs medisiner som gir fallrisiko)	Indikasjon på flere sykdommer. Vurder behov for legemiddelgjennomgang, tjenesteansvarlig / farmasøyt / fastlege
**1.11**	Balanse- og eller gangproblemer?	Diskuter behov for oppfølging av fysioterapeut eller om andre tiltak bør iverksettes, hjelpemidler?
**1.12**	Er vedkommende i stand til å reise seg fra en stol?	
**1.13**	Problemer med føtter / sko/ smerter/ hevelse i beina?	Vurder fotpleier, innkjøp av nye sko, henvis fastlege ved smerter hevelse i beina
**1.14**	Alkohol- rusmiddelbruk	Vurder om dette kan være årsak til fall
	**Risiko i hjemmet**	
**2.1**	Er boligen rotete, overmøblert, løse tepper, ledninger, høye terskler, dårlig belysning?	Er boligen allerede tilrettelagt?<br>Kontakt ergoterapeut for tilrettelegging i samarbeid med personen og eventuelt pårørende.
**2.2**	Trygt baderom inkl. adkomst?	
**2.3**	Trygt inngangsparti inne og ute? Glatte trapper / gulv?	
**2.4**	Annet?	
	**Sosialt nettverk**	
**3.1**	Pårørende/ venner som kan bidra?<br>Deltakelse i aktiviteter utenfor hjemmet?	Samarbeid med personen og eventuelt pårørende. Hvis lite sosialt nettverk og redusert aktivitet, sjekk mulighet i bydel f. eks med seniorveileder som har god oversikt over ulike tilbud. Redusert aktivitet utenfor hjemmet, kan være tidlig tegn på funksjonstap.
