# Supplementary material for: Falls prevention and management for older adults in home care services in Norway: a retrospective patient record review
Source: Eur Geriatr Med. 2025 May 4;16(3):1013–23. doi: 10.1007/s41999-025-01224-w (PMC12174202; doi:10.1007/s41999-025-01224-w)
Supplement: Supplementary file 2 — Supplementary file2 (PDF 160 KB) Steps to promote thorough and consistent data collection [file 41999_2025_1224_MOESM2_ESM.pdf]

Online resource 2. Steps taken to promote thorough and consistent data collection.

Falls prevention and management for older adults in home care services in Norway: A retrospective patient record review
European Geriatric Medicine

- Rune Solli, Department of Rehabilitation Science and Health Technology, Faculty of Health Sciences, OsloMet - Oslo Metropolitan University, Oslo, Norway. E-mail: RuneSoll@OsloMet.no.
- Nina Rydland Olsen.
- Linda Aimée Hartford Kvæl.
- Kristin Taraldsen.
- Therese Brovold.

Table S1. Steps taken to promote a thorough and consistent data collection process, adapted from Prusaczyk et al. [1].

Stage	Description
**Before data collection**	
Operationalising variables.	Risk factors for falling and fall prevention interventions were categorised according to recommendations [2,3], checklist items (Online resource 1), and based on feedback from data collectors.
Communicating with the city districts and university privacy consultants.	We consulted with leaders, clinicians, and data collectors in each city district on data collection, including identification of participants and relevant electronic health records (EHRs). Privacy consultants at the university helped apply for ethical approval, conduct risk assessments, create data transfer agreements, and write letters of information to participants.
Piloting the data extraction template.	The data collection template was piloted and finalised using the EHRs from the first five participants, in cooperation with one data collector.
**During data collection**	
Using free-text fields.	We created free-text fields in the data collection tool (Online resource 3). These fields were used for data abstraction, i.e., to manually record new and/or unexpected information related to the documentation of risk factors, referrals, or interventions to prevent falls.
Allowing for variation in the time spent collecting data.	The time needed for data collection varied from one month in one city district to five months in another, due to competing tasks.
**After data collection**	
Communicating with the city districts.	We maintained a continuous dialogue with the city districts when preparing data for analysis to correct errors and fill in empty data fields, and when interpreting results. For instance, a data collector helped extract allocated service time for participants where this information was missing.

References

1. Prusaczyk, B.; Fabbre, V.; Carpenter, C.R.; Proctor, E. Measuring the Delivery of Complex Interventions through Electronic Medical Records: Challenges and Lessons Learned. *eGEMs (Generating Evid. Methods to Improv. patient outcomes)* **2018**, *6*, 10, doi:10.5334/egems.230.
2. Schelbred, A.-B.; Smedshaug, G.; Haugen, I.K.; Nordvik, J.E.; Belander, O.; Melby, A.K.I.

Fallforebygging Hos Eldre - Nasjonale Faglige Råd; Oslo, 2024;
3. Montero-Odasso, M.; van der Velde, N.; Martin, F.C.; Petrovic, M.; Tan, M.P.; Ryg, J.; Aguilar-Navarro, S.; Alexander, N.B.; Becker, C.; Blain, H.; et al. World Guidelines for Falls Prevention and Management for Older Adults: A Global Initiative. *Age Ageing* **2022**, *51*, doi:10.1093/ageing/afac205.
