# Supplementary material for: Falls prevention and management for older adults in home care services in Norway: a retrospective patient record review
Source: Eur Geriatr Med. 2025 May 4;16(3):1013–23. doi: 10.1007/s41999-025-01224-w (PMC12174202; doi:10.1007/s41999-025-01224-w)
Supplement: Supplementary file 3 — Supplementary file3 (PDF 428 KB) Data collection tool [file 41999_2025_1224_MOESM3_ESM.pdf]

Online resource 3. Data collection tool.

Falls prevention and management for older adults in home care services in Norway: A retrospective patient record review
European Geriatric Medicine

Rune Solli, Department of Rehabilitation Science and Health Technology, Faculty of Health Sciences, OsloMet - Oslo Metropolitan University, Oslo, Norway. E-mail: RuneSoll@OsloMet.no.
Nina Rydland Olsen.
Linda Aimée Hartford Kvæl.
Kristin Taraldsen.
Therese Brovold.

The first row shows the variables used during data collection. Starting from the second row, each subsequent row is used to fill in the respective information for a particular patient.

Deltaker-ID	Kjønn	Alder	Bosituasjon	Hvilke_tjenester	Ant_timer_hjemmesykepl_per_uke	Ant_timer_PB_per_uke	ADL-skår

Ant_fall_siste_år	Ant_diagnoser	Ant_medisiner	Bruker_innlagt	Sjekkliste_brukt	Yrkesgr_brukte_sjekkliste

Kartlagt_risiko_hos_bruker	Akutt_sykd	ABCDE	NEWS	SAFE	Vurd_endr_funksjon	Svimmelhet	Besvimelse

Ant_fall_siste_3_mnd	Brudd_ifm_fall_siste_10_år	Sykd_øke_fallris	Demens	Kogn_svikt	Neds_syn	Neds_hør	Vekttap

Årsak_vekttap	Hyppige_toalettbe	Årsak_hyppige_toalettb	Polyfarmasi	Balanseprob	Gangprob	Reise_seg_stc	Prob_fot

Prob_sko	Smerte_hevelse_ben	Alkoholbruk	Rusbruk	Ort_blodtr	Hjerterytme	Urinprøve

Fritekst_Individuelle_risikofakt	Kartlagt_risiko_i_hjem	Rotete_bolig	Overmøbl	Løse_tepper	Løse_ledn

Høye_terskler	Dårlig_belys	Trygt_baderom	Trygt_inngangsp	Glatte_trapp_gulv	Annet_Omgivelser	Pårørende_venner

Delt_akt_utenf_hjem	Beskrivelse_fall	Årsak_fall	Burde_pårør_	Disk_tverrfag_dato	Kont_fysio	Kont_fastlege

Kont_ergo	Kont_TA_sykepl_helsefag	Kont_sosionom	Kont_seniorveilede	Kont_frisklivssentral	Kont_fotpleier

Kont_ernær	Kont_optiker	Kont_farmasøyt	Kont_øyelege	Endring_i_tiltaksplan	Kont_annet

Fritekst_Tiltak_henvisning_kontakt	Tilt_trening	Tilt_tilrett_hjem	Tilt_ernæring_væsk	Tilt_legemiddelgjenn	Tilt_iverksatt_av_fastleg

Tilt_nye_sko	Tilt_samarb_pårørende	Tilt_syn_hørsel	Tilt_annet	Hjelp_rullator	Hjelp_annet	Fritekst_Hjelpem
