# Supplementary material for: Falls prevention and management for older adults in home care services in Norway: a retrospective patient record review
Source: Eur Geriatr Med. 2025 May 4;16(3):1013–23. doi: 10.1007/s41999-025-01224-w (PMC12174202; doi:10.1007/s41999-025-01224-w)
Supplement: Supplementary file 4 — Supplementary file4 (PDF 158 KB) Detailed results [file 41999_2025_1224_MOESM4_ESM.pdf]

Online resource 4. Detailed results.

Falls prevention and management for older adults in home care services in Norway: A retrospective patient record review

European Geriatric Medicine

- Rune Solli, Department of Rehabilitation Science and Health Technology, Faculty of Health Sciences, OsloMet - Oslo Metropolitan University, Oslo, Norway. E-mail: RuneSoll@OsloMet.no.
- Nina Rydland Olsen.
- Linda Aimée Hartford Kvæl.
- Kristin Taraldsen.
- Therese Brovold.

Table S2 Adherence to recommendations on fall risk assessment and fall prevention interventions. This table provides a comprehensive list of the city districts' documentation of the fall risk factors and interventions to prevent falls recommended by the WFG2022, grouped by fall risk.

**Fall risk factors and fall prevention interventions**	**Intermediate fall risk patients*, n = 54**		**High fall risk patients*, n = 171**	
**n (%)**				
Received a multifactorial fall risk assessment**	33	(61)	120	(70)
Balance, gait, muscle strength	27	(50)	99	(58)
Of these, an intervention of exercise or physiotherapist†	12	(22)	76	(44)
Footwear and foot problems‡	16	(30)	44	(26)
Of these, an intervention for footwear or feet‡	1	(2)	4	(2)
Concerns about falling§	-	-	-	-
Of these, an intervention to address concerns about falling§	-	-	-	-
Dizziness	11	(20)	46	(27)
Vision or hearing loss¶	21	(39)	76	(44)
Of these, an intervention of vision/hearing optimisation¶	1	(2)	4	(2)
Cognition††	13	(24)	62	(36)
Behaviour (alcohol or drug use)	8	(15)	48	(28)
Orthostatic hypotension	11	(20)	47	(27)
Urinary incontinence	12	(22)	36	(21)
Cardiovascular disorders#	8	(15)	51	(30)
Contributing diseases##	28	(52)	111	(65)
Osteoporosis+	9	(17)	37	(22)
Of these, osteoporosis treatment!	0	(0)	0	(0)
Parkinson's disease	18	(33)	74	(43)
Depressive disorders	-	-	-	-
Polypharmacy!!	18	(33)	63	(37)
Of these, medication intervention	1	(2)	11	(6)
Nutritional status	16	(30)	52	(30)
Of these, nutritional intervention	2	(4)	18	(11)
Vitamin D§	-	-	-	-
Environmental risk	21	(39)	85	(50)
Of these, environmental intervention	11	(20)	57	(33)
Used the checklist during assessment	15	(28)	58	(34)

Received a multifactorial fall prevention intervention‡‡	10 (19)	68 (40)
**Median (min – max)**		
Number of fall risk factors assessed per patient	3 (0 – 16)	7 (0 – 16)
Number of fall prevention interventions provided per patient	1 (0 – 5)	2 (0 – 6)

The table shows how many patients received the indicated fall risk assessment, and of those, how many received indicated intervention (indicated in grey rows). No intervention underneath a risk factor means that no intervention was systematically initiated.
* Intermediate fall risk: 1 fall past 12 months and ADL score < 3 and no admission to an institution following the fall. High fall risk: ≥ 2 falls past 12 months or ADL score ≥ 3 or an admission to an institution following the fall.
** An assessment of ≥ 2 modifiable fall risk factors.
† An intervention including physical exercise or referral to physiotherapist.
‡ Assessment of issues with feet or footwear. Management included advice about footwear and ordering new shoes, or a referral to a pedicurist.
§ Not systematically assessed or managed following a fall.
¶ May include advice about glasses, or referral to optician or ophthalmologist.
†† Dementia or cognitive impairment.
# Includes assessment of hearth rhythm and referral to general practitioner.
Includes assessment of diseases that increase fall risk, ABCDE, NEWS 2, SAFE, urine test, pain or swelling in legs or feet, and referral to general practitioner.
+ Assessment of fall-related fractures during the past 10 years.
! No information about osteoporosis assessment or treatment available in the patient records.
!! Use of four or more prescription medications.
‡‡ An intervention consisting of ≥ 2 components, one of which was exercise or referral to a physiotherapist.
